# Supplementary material for: Identification of photoperiod-induced specific miRNAs in the adrenal glands of Sunite sheep (Ovis aries)
Source: Front Vet Sci. 2022 Jul 22;9:888207. doi: 10.3389/fvets.2022.888207 (PMC9354845; doi:10.3389/fvets.2022.888207)
Supplement: Supplementary file 1 [file Data_Sheet_1.ZIP › Supplementary file/miRDeep2 file.zip!/miRDeep2 file/Know_Structure/SPLP42A1/oar-mir-374b.pdf]

miRBase precursor	: oar-mir-374b
Total read count	: 26397
oar-miR-374b read count	: 25924
remaining reads	: 473

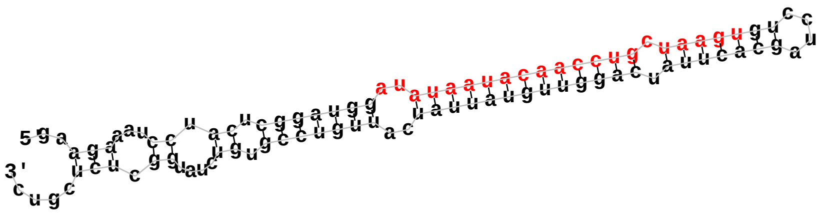

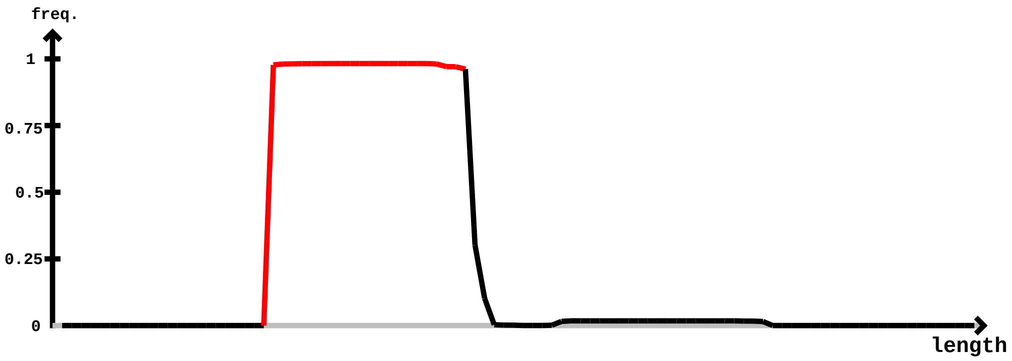


```
                    oar-miR-374b
5'- gaagaaauccuacucggauggauauaauacaaccugcuaaguguccuagcacuuaucagguuguauuaucauuguccgugucuauggcucucguc   -3'    exp
    ((((..((...((.(((((((..(((((((((((((.(((((((....))))))).)))))))))))))..))))))).))..))..)).))...    reads     mm     sample
    .....................auauaauGcaaccugcu.........................................................    1         1      seq
    .....................auauaauacaaccugcA.........................................................    3         1      seq
    .....................auauaauacaaccugcu.........................................................    34        0      seq
    .....................Cuauaauacaaccugcu.........................................................    5         1      seq
    .....................auauaauacaaccugcua........................................................    253       0      seq
    .....................auauaauacaaccugcuaa.......................................................    19        0      seq
    .....................auauaauacaaccugcuaag......................................................    239       0      seq
    .....................auauaauacaaccugcuaagu.....................................................    17383     0      seq
    .....................auauaauacaaccugcuaagug....................................................    5264      0      seq
    .....................auauaauacaaccugcuaagugu...................................................    2563      0      seq
    .....................auauaauacaaccugcuaaguguc..................................................    23        0      seq
    .....................auauaauacaaccugcuaagugucc.................................................    3         0      seq
    .....................auauaauacaaccugcuaaguguccu................................................    5         0      seq
    .....................auauaauacaaccugcuaaguguccuagc.............................................    3         0      seq
    .....................auauaauacaaccugcuaaguguccuagcacu..........................................    1         0      seq
    ......................uauaauacaaccugcua........................................................    1         0      seq
    ......................uauaauacaaccugcuaagu.....................................................    21        0      seq
    ......................uauaauacaaccugcuaagug....................................................    7         0      seq
    ......................uauaauacaaccugcuaagugu...................................................    48        0      seq
    ......................uauaauacaaccugcuaaguguc..................................................    8         0      seq
    ......................uauaauacaaccugcuaagugucc.................................................    1         0      seq
    ......................uauaauacaaccugcuaaguguccu................................................    3         0      seq
    .......................auaauacaaccugcuaagu.....................................................    2         0      seq
    .......................auaauacaaccugcuaagug....................................................    2         0      seq
    .......................auaauacaaccugcuaagugu...................................................    2         0      seq
    .......................auaauacaaccugcuaaguguc..................................................    3         0      seq
    .......................auaauacaaccugcuaagugucc.................................................    2         0      seq
    .......................auaauacaaccugcuaaguguccu................................................    2         0      seq
    ........................uaauacaaccugcuaagu.....................................................    1         0      seq
    ........................uaauacaaccugcuaagugu...................................................    1         0      seq
    ........................uaauacaaccugcuaagugucc.................................................    2         0      seq
    ........................uaauacaaccugcuaaguguccu................................................    16        0      seq
    ........................uaauacaaccugcuaaguguccuagc.............................................    1         0      seq
    .........................aauacaaccugcuaaguguccu................................................    5         0      seq
    .........................aauacaaccugcuaaguguccuag..............................................    1         0      seq
```

```
                     oar-miR-374b

gaagaaauccuacucggauggauauaauacaaccugcuaaguguccuagcacuuaucagguuguauuaucauuguccgugucuauggcucucguc
..........................auacaaccugcuaaguguccu................................................   1    0    seq
..........................auacaaccugcuaaguguccua...............................................   1    0    seq
..........................auacaaccugcuaaguguccuag..............................................   1    0    seq
...........................uacaaccugcuaagugA...................................................   1    1    seq
.................................................cacuuaucagguuguauuauc.........................   2    0    seq
..................................................acuuaucagguuguauuau..........................   1    0    seq
..................................................Ccuuaucagguuguauuauc.........................   1    1    seq
..................................................acuuaucagguuguauuauc.........................   11   0    seq
..................................................acuuaucagguuguauuauca........................   2    0    seq
..................................................acuuaucagguuguauuaucau.......................   13   0    seq
...................................................cuuaucagguuguauuauc.........................   5    0    seq
...................................................cuuaucagguuguauuauca........................   2    0    seq
...................................................cuuaucagguuguauuauAau.......................   1    1    seq
...................................................cuuaucagguuguauuaucau.......................   27   0    seq
...................................................cuuaucagguuguauuaucauu......................   343  0    seq
...................................................cuuaucagguuguauuaucauug.....................   3    0    seq
....................................................uuaucagguuguauuaucau.......................   2    0    seq
....................................................uuaucagguuguauuaucauu......................   52   0    seq
```
